# Statistical Approach to Estimating Audience from MAC-Randomized WiFi Probe Requests

**DOI:** 10.3390/s22228679

**Published:** 2022-11-10

**Authors:** Feifei Yang, Iness Ahriz, Bruce Denby

**Affiliations:** 1 Aleia, 75008 Paris, France; 2 Institut Langevin, ESPCI Paris, PSL University, CNRS, Sorbonne Université, 75005 Paris, France; 3 C.N.A.M. (National Conservatory of Arts and Crafts), 75003 Paris, France

**Keywords:** audience monitoring, GDPR, IoT, MAC randomization, probe request, WiFi

## Abstract

In the past few years, the ability of wireless network operators to monitor audience using control frames emitted by client devices has been compromised, both by legislation treating client MAC addresses as private information and by the difficulty of distinguishing genuine client frames from those arising from the Internet of Things or from certain enhanced services. Here, a deterministic model, based on characteristics of human activity and on seasonal trends, is used to reveal underlying client statistics in raw MAC-randomized WiFi Probe Request data. The method proposes a candidate conversion factor, *X*, between probe request counts and the client population, which offers plausible predictions on real-world datasets.

## 1. Introduction

For a number of years, it has been standard industry practice to leverage the fixed client MAC address contained in WiFi Probe Requests (PR) as a means of counting clients, both for commercial and security purposes. With the advent of the European General Data Privacy Regulation (GDPR) in 2016, treating MAC addresses as personal information, followed by a progressive strengthening of these regulations in the ensuing years and a proliferation of similar initiatives in other countries, the search for new techniques of audience monitoring has become active.

The *de facto* tool for enforcing GDPR in WiFi networks is the randomization of client MAC addresses performed by the majority of mobile device operating systems in use today. Pioneering studies of a detailed PR frame structure, e.g., [1,2,3], have enabled possible workaround solutions. One line of attack is to exploit existing privacy flaws to perform MAC de-randomization [4,5]; however, a consensus is emerging that data privacy is here to stay and that adopted solutions must be immune to randomization [6,7,8,9,10]. 

It is tempting to handle randomized MACs by leveraging clients with fixed MACs—those who have subscribed to the local WiFi service or whose operating systems simply do not perform randomization—under the assumption that the PR emission statistics of these two classes will be similar [7,8]. This hypothesis, however, is challenged by growing numbers of Internet of Things (IoT) devices and by clients employing enhanced services (ES), e.g., Peer-to-Peer (P2P), both of which generate very atypical PR statistics as compared to genuine client profiles. Estimating audience from randomized MACs alone is thus both challenging and increasingly necessary, and as such, solutions—even approximate ones—are very much in demand.

To date, most approaches to the problem (e.g., [8,9,10,11]) require fine-grained time-domain interaction with PR records and are computationally intensive. They are typically developed in monitored test runs involving the close interplay between bespoke network hardware and a laboratory software development team. Invariably, some type of “ground-truth” calibration technique, such as camera systems or special sensors that can reliably count customers, is required, which can be very costly to install and operate. 

The present work proposes a simple statistical approach based solely on discovering underlying client statistics. It leverages daily PR counts and common aspects of client activity and seasonal trends to produce an approximate scale factor, *X*, to convert raw PR counts directly to client population estimates. Based exclusively on randomized MAC clients, it is inherently privacy-preserving and avoids problems of IoT and ES clients. The method furthermore can be applied to the real world, a posteriori datasets acquired in commercial WiFi networks that do not include a supplementary ground truth system. Indeed the proposed method is believed to be the first to enable audience monitoring based solely on randomized MAC PR statistics without requiring an additional ground truth technique. 

Section 2 introduces the datasets and definitions used, followed by a description of the method and a summary of the experimental results. In Section 3, five separate “validations” demonstrate that the proposed method’s results are reasonable. Three of these validations are based on complementary analyses of our own datasets, while the remaining two provide comparisons with other currently existing methods. In the final section, we balance the originality and simplicity of our approach against its current shortcoming and limits of applicability and provide possible routes for evolving beyond them.

## 2. Materials and Methods

### 2.1. Datasets and Definitions

The datasets provided to our group consist of timestamped PR records from several public outdoor WiFi networks in France. The data are not public, and site names have been anonymized. Please see the data availability statement at the end of the article for details. 

For the present article, segments of data from three cities and a campground were explored. Data were recorded in 2020 and 2021. Some of the city data were split into two periods according to whether COVID-19 confinement was in effect or not. Overall, six files were created from the raw database, as detailed in Table 1. The cities studied cover a wide range of sizes and populations, which, as will be seen later, allows us to test our approach on PR counts varying over nearly an order of magnitude. The inclusion of the campground dataset, to be discussed in Section 3, which has logistics quite different from those of a city, further helps to ensure the general applicability of the proposed method. 

A separate file stores connection detail records of clients who have at some time formally signed in to the WiFi service. In accordance with the GDPR, client MAC addresses were not retained during data acquisition and, in the files provided to us, are replaced by anonymized hash-coded strings. It is important to note that a fixed MAC address will always hash to the same string, while for randomized MACs, the hash code will be different for each PR a client emits.

Although the timestamps on each file record allow us to choose any desired time step, for the purposes of this article, PRs have been aggregated into daily totals. Then, by counting the number of times in a one-day period that a distinct anonymized MAC is seen and comparing it to a threshold, and by taking into account the aforementioned connection details file, we may define a day’s PRs as belonging to one of the three classes below. Since connected clients are separated out first, the classes are mutually exclusive.

1. Client Randomized, CR
  The MAC string is seen from 1 to “threshold” times.
2. Client Fixed, CF
  The MAC string is seen more than “threshold” times.
3. Client Connected, CC
  The MAC string appears in the connection details file.

**Figure 1:**
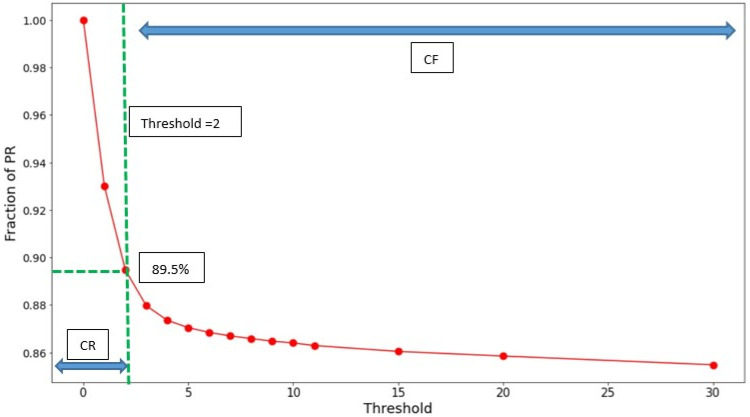
Behavior of the number of PR retained versus the threshold used to define the CF and CR classes. A threshold of 2 is chosen so that anonymized MAC addresses seen 1 or 2 times are considered to belong to the class of randomized clients, or CR, while those appearing more frequently are considered fixed MAC, or CF.

If CR clients’ MAC addresses are truly randomized, every CR MAC address should be unique, and we would have a threshold = 1. An initial study, however, indicated the situation is more complicated. Figure 1 shows the fraction of total PR retained as a function of the threshold for a typical dataset. For thresholds from 30 down to about 4, the percentage of PR retained is almost independent of the threshold, meaning that most MAC addresses are seen many times—this is the CF class. Thresholds of 2 or 3 are somewhat of a gray area. The PRs retained could correspond to CRs whose MAC randomization was not perfect—techniques are, after all, vendor dependent and sometimes based on specific time windows. Or these could be individual CFs that contribute little traffic. Too high a threshold presents the danger of “polluting” the CR class with IoT devices, as explained in the next section. In contrast, too low a threshold might unnecessarily reduce the efficiency of CR. Most likely, both scenarios contribute to PRs in this region. As a compromise, a threshold of 2 was chosen to separate the CR and CF classes. Picking a larger threshold has very little impact on the numerical results obtained; however, choosing a threshold of 1—which we believe is too restrictive—reduces the *X* conversion factors we measure by about 25%, which is a source of uncertainty but does not fundamentally alter the significance of the results that will be presented. 

### 2.2. OUI, IoT, and General Trends

**Figure 2:**
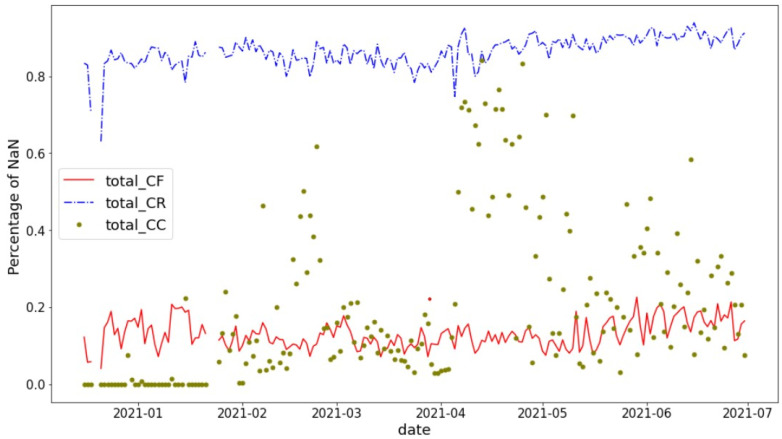
Percentage of NaN OUIs by client class for city-A.

Despite the randomization of the MAC, its first 3 bytes, called the Organizationally Unique Identifier, or OUI, are retained and become part of the PR entry. The OUI is intended to provide information on the manufacturer of the WiFi network card producing the PR, available by interrogating a public OUI web repository. OUI usage, however, is unregulated and voluntary, and in practice, the OUI request will often return NaN, i.e., undefined. In the data sets studied, the percentage of NaN OUI responses was found to depend heavily upon the client class defined in Section 2.1, as illustrated in Figure 2.

**Figure 3:**
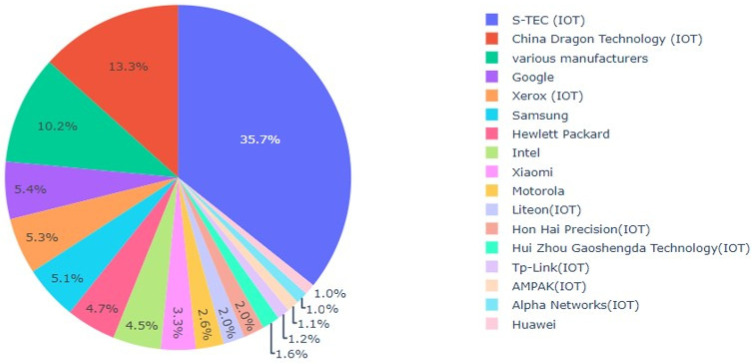
Percentage of different CF OUI manufacturers on a typical day in city A.

Displayed are the percentages of NaN OUI responses for CR, CF, and CC client classes for PR in city 1 over a 6-month period in 2021. The figure shows that PRs of randomized clients, i.e., originating from GDPR-compliant operating systems, also tend to mask device manufacturer information. Fixed client OUIs are most often available, as expected; however, this observation comes with a caveat. Figure 3 shows that OUI responses include a baffling array of manufacturers that are sometimes difficult to identify clearly. A detailed investigation suggests that up to 80% are manufacturers of IoT devices, such as lighting, heating, cameras, etc., have a network behavior that will be quite distinct from true client devices. Connected clients exhibit mixed OUI behavior. In addition, CC is susceptible to containing ES clients, which we will return to in Section 3. These observations provide a strong motivation for basing GDPR-compliant crowd estimation solutions exclusively on randomized MAC clients.

### 2.3. Description of the Method

**Figure 4:**
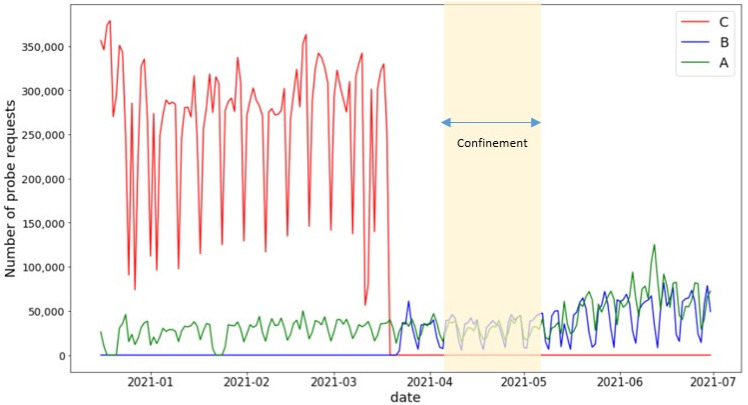
Daily CR PR counts. Period 1 in city-B, or city-B1, was a COVID–19 confinement period.

Figure 4 portrays the daily randomized MAC PR counts in the three cities over a period of several weeks. The figure shows the weekly periods punctuated by weekends, occasional departures from regularity corresponding to holidays, and some irregular periods. Scale shifts relating to periods of COVID-19 confinement, and apparent small upward drifts, most likely seasonally related, can also be discerned. The key concept of the proposed method is to exploit the observed periodic structure to reveal the underlying statistics of the PR data.

**Figure 5:**
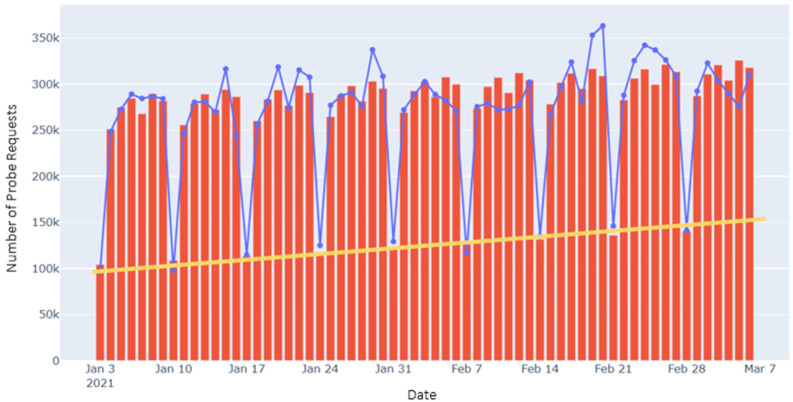
Example graph of CR data versus time, with the model, superimposed, for city-C2. The blue line is the raw PR data, and the red bars are the fitted model. A yellow line indicates the linear trend term of the model.

To implement the procedure, we first choose an interval where clean periodicity is apparent in the PR data for a particular site and consider the data as a set of experiments repeated over several weeks. A model of the data is then constructed in the form of a repeated weekly template composed of mean numbers of PRs for each of the 7 days of the week, to which we add a linear trend term to account for possible seasonal drifts. The model parameters are determined by minimizing, with respect to these parameters, the summed squared deviations of the data from the mode, i.e., by performing a least mean squares fit of the model to the data. An example of the model (red bars) superimposed on raw PR counts (blue curve) for city-C is shown in Figure 5, where the linear trend term is also explicitly indicated by a yellow line. The proposed algorithm, described below, is based on interpreting the squared deviations of the data points from the model, along with some additional assumptions.

To derive the algorithm, let us consider a site equipped with WiFi access points capable of capturing PRs from telephones carried by visitors to the site, also known as clients. The experiment has a duration *T* in some units—in this article, we will usually take *T* equal to 1 day—and the total number of visitors during the period is *A*. We let *t_b_* < *T*, with *b* ∈ {1…*A*}, be the duration of client *b*’s stay at the site, and *x_b_* the mean number of PR emitted by client *b*’s telephone during a period of duration *T*. Indeed, it is well known that telephone PR emission rates vary widely depending on the telephone operating system used and the current state of the device, as discussed for example in [1]. We will then have, for the total number of PR emitted, *P*:

(1) $$P=\sum_{b=1}^{A}\frac{x_{b}}{T}t_{b}=\frac{A}{T}\sum_{b=1}^{A}\frac{x_{b}t_{b}}{A}=\frac{A}{T}\langle x_{b}t_{b}\rangle ,$$

where < > denotes the mean of the quantity within the brackets. Since we would expect the duration of a client’s stay at a site to be independent of the PR emission rate of his or her telephone, we may use the law of the mean of the product of two independent distributions to write:

(2) $$P=\frac{A}{T}\langle x_{b}\rangle \langle t_{b}\rangle =\left(\frac{\langle x_{b}\rangle }{T}\right)\left(A\langle t_{b}\rangle \right)$$

(3) P≡XC

where we now implicitly take *T* to be one day, interpreting *X* as the mean, over all telephones, of the number of PR emitted by one client over a full day, and *C* the number of effective client days in the experiment. For example, *C* = 2, that is, 2 client-days, could correspond to two clients both present for an entire day, four clients each present for half a day, etc. As another example, if we predict *C* = 500 clients for one day, of which 250 remain the entire day, while the remaining 250 are progressively replaced by 250 different clients, we still have *C* = 500, while *A* = 750.

One may ask why we choose to predict *C* in client days instead of *A*, the actual number of different clients. Indeed, due to MAC randomization, we cannot identify individual clients and thus only have access to a “bulk” figure, such as *C*. The choice of preferring *A* or *C*, however, is application dependent. For personal safety applications, for example, such as the occupation capacity of a building or a site, the essential figure is *C*, the number of persons present in a certain time window, not the total number of persons who passed through the site during the period. On the other hand, if one is interested in the number of tickets sold at a site, independently of how much time each visitor spends there, *A* is the more appropriate number. When local ground truth data—for example, gate receipts—are available, methods exist for estimating *A* from *C* on average (e.g., [4,7]), but these are not within the scope of this paper.

At this stage, we have a means of estimating the number of client days from the number of probe requests via a simple multiplicative factor *X*. It now remains to propose a method of obtaining *X* from the data. The technique proposed is based on the squared deviations of the PR data from the model. Deviations from the mean number of PR for a particular day will have as their source random fluctuations in the numbers of clients, *C*, of course, but also, conceivably, time-dependent fluctuations in the value of *X*. To quantify this, we make use of the expression for the variance of the product distribution, in this case, *P* = *XC*, which gives:

(4) P=XC

(5) $$\sigma_{P}^{2}=\sigma_{C}^{2}X^{2}+\sigma_{C}^{2}\sigma_{X}^{2}+\sigma_{X}^{2}C^{2}$$

(6) $$\frac{\sigma_{P}^{2}}{P}=\frac{\sigma_{C}^{2}X}{C}+\frac{\sigma_{C}^{2}\sigma_{X}^{2}}{CX}+\frac{\sigma_{X}^{2}C}{X}$$

where the *σ*^2^ terms are the variances of the parent and product distributions. As *C* arises from a counting experiment over a fixed time interval, we expect it to have a Poisson distribution. Since the variance of a Poisson distribution is equal to its mean, we may now write:

(7) $$\frac{\sigma_{P}^{2}}{P}=X+\frac{\sigma_{X}^{2}}{X}+\frac{\sigma_{X}^{2}C}{X}$$

(8) $$\frac{\sigma_{P}^{2}}{P}\approx X+\frac{\sigma_{X}^{2}C}{X}$$

(9) $$\frac{\sigma_{P}^{2}}{P}\approx X+\frac{\sigma_{X}^{2}}{X^{2}}P$$

where in (8), we have assumed that *C* >> 1, and in (9), we replace *C* with *P*/*X* in order to obtain an equation involving only *X* and *P*. The left-hand side of (9) is the Variance to Mean Ratio of *P*, also known as its Fano factor, which, when superior to one, reveals correlations in the deviations about a mean value, as would be expected for a population of clients each emitting multiple PRs. We see that here it provides a simple measure of the mean conversion factor *X* from clients to PR, along with an additional term, due to possible variability in the value of *X*, that may grow with the number of PR. We now proceed to study the application of this equation to our randomized MAC (i.e., CR) PR data.

### 2.4. Results

**Figure 6:**
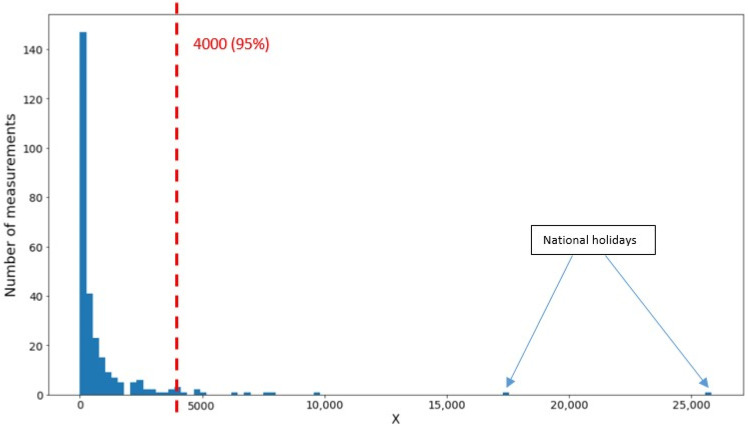
Cut used to exclude outliers from the *X* distribution.

Using a model of the type illustrated in Figure 5, we now calculate the variance of *P* from the scatter of the data points about the constructed template and use it to calculate an *X* for each site. Since it arises from a variance, the calculation is sensitive to outliers. An outlier cut was designed to handle this problem by combining the *X* distributions from all sites into a single plot and choosing an appropriate threshold. The resulting distribution is shown in Figure 6. A drawback to this approach is that the *X* distributions might be different for the different sites; however, as the statistics in our samples are somewhat limited—one measurement per day over a period of a few weeks—the combined distribution seems a more reliable way to proceed. The resulting cut, placed at *X* = 4000, beyond the body of the distribution and at the beginning of the “tail,” preserves 95% of the data. The figure shows two outliers corresponding to French national holidays, which clearly must be removed from the sample. It also seems likely that there exist less “formal” sources of large correlated client population fluctuations—transit strikes or outages, social movements, etc.—that also contribute to the tail of the distribution.

**Figure 7:**
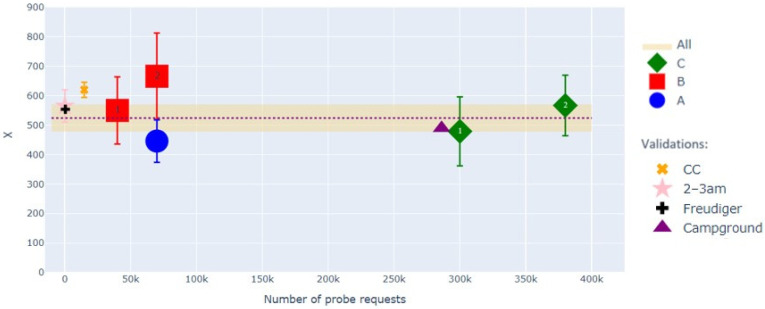
Value of *X* for the 5 datasets for cities A, B, and C, versus *P*. Validation points shown in the figure are discussed in Section 3; for better readability, an additional validation, using [9], is not shown in the graph but is discussed in detail in Section 3. Symbol labelled “Freudiger” refers to [1].

Removing the outlier cut completely would be unreasonable and skew the results dramatically. Simply adjusting the position of the cut would introduce a shift in mean *X* approximately equal to the current cut value, 4000, times the fraction of the data points gained or lost through the shift. As the current cut retains 95% of the data, a moderate change in its position might involve 1% or 2% of the data, thus resulting in a shift of the mean *X* by 40 to 80, which is comparable to the estimated standard deviations of *X* at the different sites, as discussed below in the context of Figure 7.

The resulting mean *X* values for the five city data sets are shown as a function of *P* in Figure 7, with the standard deviation of each measured mean *X* indicated by an error bar. The figure shows that within the precision of the method, *X* remains relatively constant over the full range of *P* values in our data sets—nearly an order of magnitude. This implies that we may, at this stage in our study, ignore the term linear in *P* in Equation (9). The validations mentioned in Figure 7 will be discussed in Section 3.

As mentioned earlier, *X* values for the different sites might be expected to differ due to site-dependent Access Point (AP) numbers, AP layouts, and propagation effects; however, at present, the statistical uncertainties appear to mask any such differences. As such, it is useful to combine the data into an average overall value and standard deviation, which is indicated by the dashed line within the yellow band at *X* = 524 ± 47 in Figure 7.

**Figure 8:**
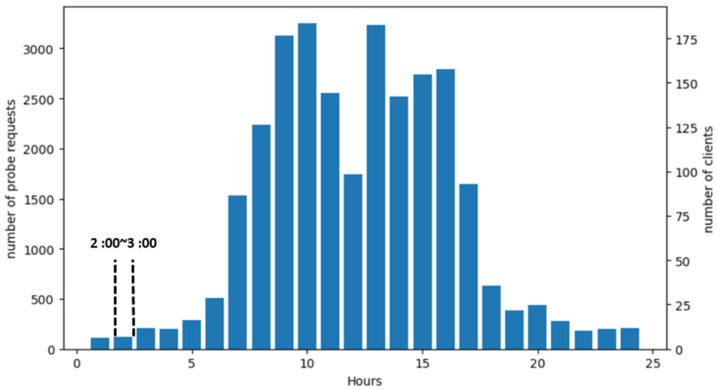
Typical hourly CR *P* (left axis) and *C* (right axis) values for city-A. The right hand axis is calibrated in client hours. The highlighted period 02:00–03:00 will be used in the validation in Section 3.

Once a satisfactory *X* is obtained, the predicted number of client days for a particular date is simply given by *C* = *P*/*X*. If it is desired to study site occupation over the course of a day, the PR timestamps can be used to partition the number of client days into client hours by dividing *X* by 24, as shown for a typical day for city-A in Figure 8. The highlighted period 02:00–03:00 in the figure will be used in one of the validations in the following section.

## 3. Validations of Results

The data used in this study come from operational outdoor WiFi systems that are not equipped with auxiliary ground truth systems, such as cameras, etc. As such, validating our results directly against such a system is impossible. It has been possible, however, to validate that the *X* values produced by our method are reasonable by bootstrapping off the statistics of our CC; by studying our CR with an alternate time window; and by deriving an *X* value for a campsite in our datasets, for which we were able to obtain head counts for a short fixed period. In addition, we compare our results with two studies from the literature that are also based on obtaining an overall rate of WiFi PR emission by telephones. All in all, five separate validations are presented in what follows.

### 3.1. Validation with CC

When MAC addresses are fixed, as for CC and CF classes, the number of actual clients can be counted, and the duration of their stays obtained from the time stamps. Thus, although we do not believe CC or CF will be useful for estimating the audience, we can nonetheless measure values of *X* for these classes and see how they compare to those obtained with our method for the CR class. Because of the difficulty of distinguishing real clients from IoT in CF, we choose to validate using CC.

**Figure 9:**
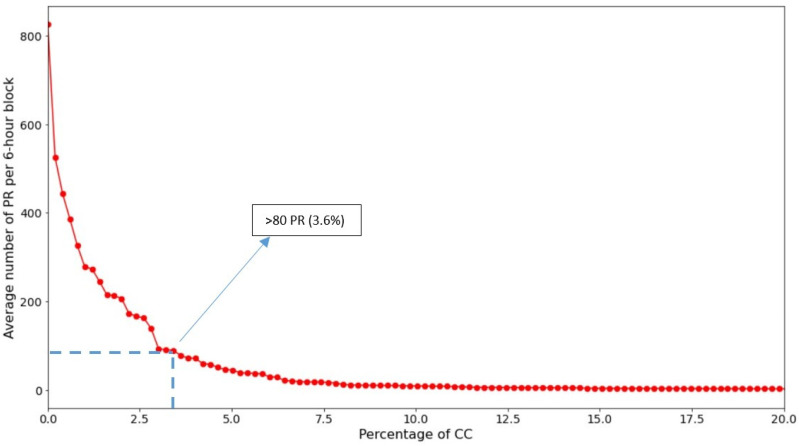
The ES-cut removes CC clients who emit an anomalously high average number of PR in six-hour blocks, consistent with P2P or other extended services. Data for city B.

As mentioned earlier, however, CC may use the subscribed WiFi connection as an anchor point for enhanced services, for example, P2P [12]; Spanning Tree Protocols [13]; chats; social networks; walkie-talkies; online games; and WiFi repeaters, etc., which create elevated PR traffic by using PRs for topology discovery; routing; or PR-encapsulated message-passing. In our study, we indeed encountered behavior consistent with ES in a small percentage of our CC clients, which has the effect of skewing *X* measurements towards higher values. The supposed ES traffic was bursty in nature in that offending clients tended to be exceptionally active in continuous blocks lasting a few hours. To address the problem, an “ES-cut” was developed, defined as emitting more than 80 PR per 6-h block, averaged over the full measuring period (i.e., including empty blocks as well). Figure 9 for city B explains how the cut value was determined. Although it is not possible to confirm absolutely that the removed clients were engaged in ES, for cities A and B, where the problem was most pronounced, we were able to confirm, using the recorded Received Signal Strength Indicator, or RSSI values of the PRs to localize their emission points, that these clients frequented a small number of fixed locations in or near city administrative buildings exclusively. This is further evidence of the non-standard nature of these clients.

Our validation quantity based on CC, *X*_CC_, is then obtained by calculating the average number of PR per CC during a day and correcting for the average daily CC occupancy computed from the PR timestamps, giving *X*_CC_ = *P*/(*A*_CC_<*t*_CC_>) with *A*_CC_ the number of CC and <*t*_CC_> their average duration of stay. Results are given in Table 2, with and without the ES-cut, along with the percentage of clients retained after the cut. As CC account only for small numbers of PR, the *X*_CC_ values of the table are reported as a single average *X*_CC_ point on the far left in Figure 7. Since *X*_CC_ is measured in a counting experiment rather than from a variance, the standard deviation of the mean *X*_CC_ values is somewhat smaller than that of the CR-based *X* values.

We stress that *X* and *X*_CC_ are not a priori expected to be identical due to the different natures of CR and CC clients, the reduced statistics of CC compared to CR, and the fact that PR emission rates are known to depend on the device state [1]. It would not be surprising to find *X* and *X*_CC_ to be similar, as is indeed the case here, lending credence to the idea that the proposed technique for obtaining *X* is indeed sensitive to the underlying statistics of CR clients.

### 3.2. Validation Based on an Alternate Template: 02:00–03:00

The second validation, this time using CR in city A, extends the Fano factor method to a new template focusing on the daily 02:00–03:00 period (see Figure 8), where PR counts are much lower and should be to be relatively independent of seasonal variations and workweek-related periodicities, due to the nocturnal nature of the timeslot selected. The cut on *X* at 4000 introduced in Section 2, scaled for the smaller time window, is also employed in calculating the *X*_02-03_ values. The results from the different sites are again combined into a single measurement, displayed near zero PR in Figure 5. The figure shows that the obtained value is consistent with *X* obtained from the weekly templates.

### 3.3. Validation from the Literature I

Freudiger et al. [1] measured PR inter-arrival periods for Samsung, iPhone, and Nexus devices in various states—screen lit, browser open, etc. One may use these measurements to deduce a global average inter-arrival period of 156 s, allowing one to obtain *X*_Freudiger_ = 554, which is reported as the “+” symbol at *P* = *X* on the left side of Figure 7. This value is technically not directly comparable to our *X*, which measures the number of PR received from a client in a genuine deployed network, not the number emitted, which could depend on local AP counts and layouts. Moreover, the variety of telephones explored in the study in [1] is presumably somewhat limited compared to that in our real-world data. However, the fact that *X*_Freudiger_ aligns reasonably with the results obtained with our method is promising.

### 3.4. Validation from the Literature II

In [9], a technique for estimating the audience from randomized PR that employs a fixed conversion factor is presented. As such, it is interesting to compare that work with our technique. As a caveat, the two methods are not really directly comparable for a number of reasons. First, [9] deals with indoor networks, whereas all our datasets are outdoor. Second, the technique in [9] requires a pre-acquisition tuning procedure based on the RSSI at the AP, used to avoid double-counting of PR, which is not possible in our case because our datasets were furnished as-is, without any possibility of performing such a tuning. Third, while it is not specified in [9] if the clients are connected or not, the university library-type environment studied in that work would seem conducive to users actually connecting to the WiFi—which, in our estimation, could give different results from CR class clients. The principal similarity of [9] to our work is the use of a constant conversion factor from clients to probe requests—called β in [9]. Still, at the same time, this is also the greatest difference compared to our method, because in [9], β is tuned to a ground truth value obtained from a camera monitoring system. In contrast, in our case, *X* is obtained purely from statistical arguments. Nevertheless, placing the tuned value β onto the same scale as *X* allows us to validate whether the two approaches seem to be measuring the same phenomenon. To do so, it is necessary to specify an additional coverage factor, κ, defined in [9], intended to take into account the geometry of the site being studied and the resulting efficiency of detecting PR. The experiments presented in [9] have κ values ranging from 1, or full coverage, to 3, partial coverage, due to areas WiFi does not reach. Since our PR are counted independently by all APs, and we have not performed any pre-tuning, it is not possible to specify an appropriate value of κ for our data, even if we might expect such a value to be in the same range as in [9]. Making that assumption and scaling β to a one-day time period then gives the range of values 960 > *X* > 320, which brackets rather nicely the values obtained on our data sets. The conclusion is that a system that was tuned to a camera-based ground truth gives *X* values rather similar to what we obtain from statistics alone. Concerning precision, the crowd sizes predicted in [9] once again, tuned on a ground truth—follow that ground truth over time to within a few percent, which is much better than the 10–20% obtained here. This result nonetheless suggests that if the precision of our statistical method can be improved, *X* should be able to give a good representation of reality without the need for a ground truth system.

### 3.5. Validation at a Campground

Obtaining ground-truth visitor counts for a city is difficult without an extensive and costly system of cameras or other sensors. Our data set, however, also includes records from a regional campground featuring on-site swimming pools, nature trails, restaurants, and other activities, and for which daily headcounts are recorded, even if they are not normally available to the public. Although we do not have a detailed record of client comings and goings at the campground for a 10-day period encompassing a long holiday weekend in May 2021, upon special request, the site superintendent reported 584 overnight visitors. During the period, these clients produced some 286,000 PR, giving *X*_campground_ = 490. This value also appears in Figure 7 and aligns rather nicely with the other cases studied. In this case, we note that CC at the campground produced only a small fraction of this number of PR. In contrast, hundreds of thousands of additional PR were produced by CF, the majority identifiable as IoT, once again underlining the importance of measuring crowd size exclusively with CR.

## 4. Discussion and Conclusions

The use of radio network signals to identify and follow wireless devices has been studied for many years using a wide variety of techniques [14,15]. It has evolved more recently into the widespread use of audience monitoring systems based on client MAC addresses contained in PRs. With the arrival of client privacy concerns and GDPR, along with the growth of IoT and new bandwidth-hungry client services, this approach is rapidly becoming untenable, thus creating a need for new privacy-preserving approaches.

Here, a Fano factor analysis of PR data from three French cities, coupled with a deterministic template model, allows us to produce an approximate measure, *X*, of the mean number of PR produced per day by randomized-MAC WiFi clients. These values are relatively stable over different sites and a range of PR volumes of roughly an order of magnitude. Validations based on connected clients, an alternate model template, a comparison with two relevant measurements from the literature, and a ground truth test at a campground site, show that predictions based on *X* are reasonable.

The technique does have some drawbacks. Based on the variance calculation, its sensitivity to outliers results in a statistical uncertainty that needs to be improved. Using a Median Absolute Deviation, or MAD, statistic, rather than a variance, could be a way forward, provided the relation between obtained MAD values and the necessary *X* value can be discovered. Furthermore, *X* can, at present, only be evaluated on PR data with daily and/or weekly periodicities. The validation based on hourly windows presented here may hold clues to applying the method to data lacking wider-scale periodicities. Finally, one may inquire whether enhanced services clients mentioned with regard to CC might also be present in CR pools. Although this seems unlikely—since a fixed internet connection is an essential component of such services—it is an eventuality to be considered. Additional perspectives include a more rigorous theoretical analysis of the statistics of PRs produced in public WiFi networks, an analysis of the variability of *X* for different sites and client populations, and a more in-depth study of the thresholding procedure for defining CR and CF classes.

Estimating audience size from randomized MACs is still a new field. The ultimate efficacy of the proposed technique will likely come over time as the result of field tests and validation opportunities. In the meantime, due to its simplicity and ready applicability in real-world systems, it should be of substantial interest.

## Figures and Tables

**Table 1:** Summary of sites studied in the article.

Site	Population	Population Density	COVID Confinement
city-A	3720	194/km^2^	no
city-B1	1870	148/km^2^	no
city-B2	1870	148/km^2^	yes
city-C1	136,250	3954/km^2^	no
city-C2	136,250	3954/km^2^	yes
Campground *	144 campsites, 11 cabins, 9 hectares		no

* Validation only.

**Table 2:** *X*_CC_ values for the different sites.

Site	*X*_CC_ Raw	*X*_CC_ ES-Cut	% Clients
city-A	802	468	99.1%
city-B1	2329	778	96.4%
city-B2	2253	948	96.4%
city-C1	743	696	99.91%
city-C2	405	378	99.97%

## Data Availability

Restrictions apply to the availability of the data used in this study, which is third-party data provided by Aleia. Requests for access to the data should be addressed to the corresponding author and must be approved by Aleia.
